# Podocyturia as a Diagnostic Marker for Preeclampsia amongst High-Risk Pregnant Patients

**DOI:** 10.1155/2012/984630

**Published:** 2012-02-21

**Authors:** Belinda Jim, Pascale Jean-Louis, Andi Qipo, David Garry, Samia Mian, Tulio Matos, Christopher Provenzano, Anjali Acharya

**Affiliations:** ^1^Division of Nephrology, Department of Medicine, Jacobi Medical Center, Albert Einstein College of Medicine, Bronx, NY 10461, USA; ^2^Department of Obstetrics and Gynecology, Montefiore Medical Center, Bronx, NY, USA; ^3^Advocate Christ Medical Center, Oak Lawn, IL, USA; ^4^Columbia University Medical Center, New York, NY, USA

## Abstract

Urinary podocyte (podocyturia) has been studied as a diagnostic marker for preeclampsia. We sought to validate its use in preeclampsia and in differentiating it from other high risk pregnancy states. We studied an obstetric population at high risk to develop preeclampsia (study group) and uncomplicated pregnancies (control group) by analyzing their urine sediment for podocytes within 24 hours of delivery. Podocytes were identified by immunohistochemistry using the podocyte-specific protein synaptopodin. Of the 56 patients who were enrolled, 29 patients were diagnosed with preeclampsia, 9 patients had hypertensive conditions such as chronic and gestational hypertension, 6 patients had Type I/II and gestational diabetes mellitus, 3 patients were classified as others, and 9 patients exhibited uncomplicated pregnancies. Podocyturia was identified in 11 out of 29 (38%) of patients with preeclampsia/eclampsia, 3 out of 9 (33%) with gestational and chronic hypertension, and 3 out of 6 (50%) with Type I/II and gestational diabetes mellitus. None of the 9 patients (0%) with uncomplicated pregnancies demonstrated podocyturia. The sensitivity and specificity of podocyturia for preeclampsia were found to be 38% and 70%. Our study showed that podocyturia does not appear to be a sensitive nor a specific marker to diagnose preeclampsia.

## 1. Introduction

Preeclampsia is a disorder affecting 5 to 10% of pregnancies and is clinically characterized by new-onset hypertension and proteinuria. Despite significant progress in our understanding of preeclampsia, there is a need for a reliable diagnostic biomarker for use in clinical practice. In the search for a biologically plausible biomarker, there have been attempts to reconcile clinical findings with pathologic changes in renal biopsies of preeclampsia. For example, renal pathology of preeclampsia in the kidney is classically described as “endotheliosis”, or swelling of endothelial cells in the glomerulus. The podocyte, a specialized visceral epithelial cell that lines and forms the slit diaphragm of the glomerular basement membrane, has traditionally been thought to be unaffected. However, more recent microscopic studies demonstrate that podocytes are structurally changed and harbor protein resorption droplets [1]. Furthermore, there is evidence that selected podocyte-specific proteins such as nephrin, synaptopodin, and GLEPP-1 are downregulated in preeclampsia, while VEGF and Flt-1 are increased [2, 3]. Garovic et al. studied the use of podocytes in the urine (podocyturia) as diagnostic markers and found podocyturia to be highly sensitive and specific for preeclampsia [4]. We sought to study the use of podocyturia to diagnose preeclampsia and differentiate it from other conditions that may have a similar presentation in a high risk pregnancy population. We discovered that podocyturia was not very sensitive nor specific in making this diagnosis. Furthermore, podocyturia was found frequently in other high risk pregnancy states such as chronic hypertension and gestational diabetes.

## 2. Materials and Methods

### 2.1. Study and Control Subjects

We recruited two groups of patients all ≥18 years of age: uncomplicated pregnant subjects (control group) and women at risk for pregnancy complications (high-risk group) as described below from the obstetric inpatient service at Jacobi Medical Center, Bronx, NY, USA. Random urine samples were obtained from the subjects within 24 hours of delivery. Inclusion criteria for high-risk group were diagnosis of preeclampsia, chronic hypertension (HTN), gestational HTN, Type I and Type II diabetes mellitus (DM), gestational DM, mixed connective tissue disease, and pregnancies with fetal chromosomal abnormalities. Exclusion criteria were patients under the age of 18 and absence of the above-mentioned diagnosis. Inclusion criteria for the control group were uncomplicated pregnancies and deliveries and absence of the above-mentioned high risk pregnancy states. Exclusion criteria for the control group were <18 years of age, preexisting high risk pregnancy states or complicated deliveries. Diagnosis of preeclampsia fulfilled the criteria of new onset of HTN with blood pressure of 140/90 mmHg after 20 weeks of gestation and proteinuria of >300 mg of protein in a 24-hour urine specimen or 1+ protein on a urinalysis sample without evidence of another cause, such as urinary tract infection or inflammation. Chronic HTN was defined as preexisting HTN or blood pressure of 140/90 mmHg before 20 weeks of gestation. Gestational DM was defined as any degree of glucose intolerance with onset of first recognition during pregnancy. This study was approved by the Internal Review Board of Albert Einstein College of Medicine.

### 2.2. Urinary Protein Quantification

Urinary protein was quantified either from a 24-hour urine sample collection or extrapolated from a random urine dipstick. For example, when only a dipstick urine protein was available, the value was extrapolated to a 24-hour value based on the following: negative protein corresponds to less than 150 mg/24 hours, trace protein corresponds to 150 mg/24 hours, 1+ corresponds to about 200–500 mg/24 hours, 2+ to 0.5–1.5 g/24 hours, and 3+ to 2–5 g/24 hours.

### 2.3. Podocyturia

Twenty mL of freshly voided urine was centrifuged at 700 g for 5 min. The sediment pellet was carefully recovered by aspirating the supernatant, washed twice with PBS and resuspended in 1 mL of PBS. Aliquots of 100 *μ*L of the resuspended sediment were centrifuged onto slides using the Shandon Cytospin 4 Cytocentrifuge (Thermo Electron Corporation, Asheville, NC), air-dried and fixed with 1 : 1 acetone/methanol for 10 minutes. The slides were immersed with PBS/1% H_2_O_2_ for 15 minutes and washed with deionized water. Subsequently, antigen retrieval was achieved by steam-heating in a solution of citrate buffer, pH 6.0, for 15 minutes and blocked with 10% horse serum in PBS and 2% BSA. Slides were incubated overnight with monoclonal mouse antihuman synaptopodin antibody at 1 : 1 dilution (gift of Dr. Peter Mundel, Massachusetts General Hospital, Boston, MA) followed by horse anti-mouse IgG at 1 : 1000 dilution (Dako Inc. Carpinteria, CA) as secondary antibody for 30 minutes. Sections were then incubated in avidin-biotin complex at 1 : 25 dilution (Vector Labs, Burlingame, CA) and developed using diaminobenzidine (DAB) as chromogen. After washing, the sections were counter-stained with hematoxylin and coverslipped. Negative controls were carried out by incubation in the absence of the primary antibody. Podocytes were identified by positive DAB staining under light microscopy.

### 2.4. Statistical Analysis

Difference in clinical variables between more than two groups was determined by Kruskal-Wallis method, differences between two groups were determined by Mann Whitney method. Analyses were performed with STATA Version 8.2 and GraphPad Prism Version 5.02 for Windows software, and results were considered statistically significant if *P* < 0.05. For test characteristics of podocyturia, sensitivity, specificity, positive predictive value, and negative predictive value were calculated for each high risk diagnosis.

## 3. Results

**Figure 1:**
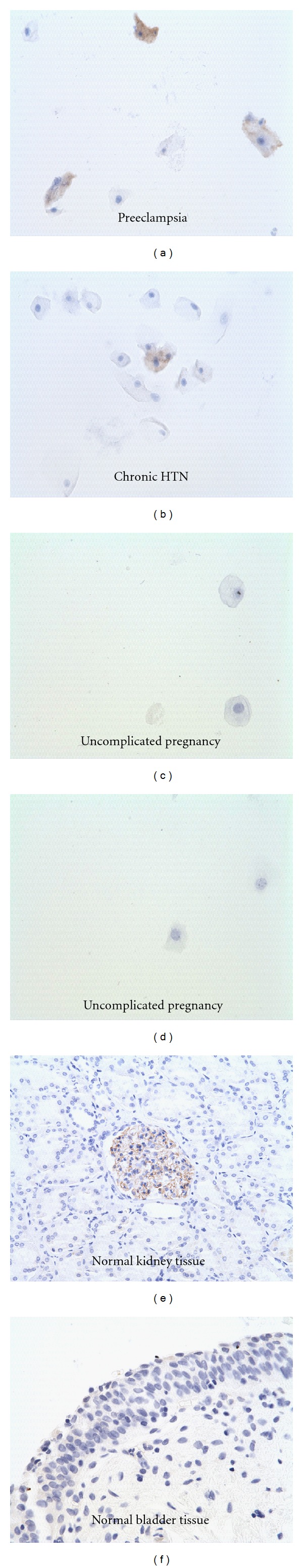
Podocyturia in high risk and uncomplicated pregnancies. (a) and (b) representative images of podocyturia identified by positive synaptopodin staining, (c) and (d) Representative images of negative staining in uncomplicated pregnancies, (e) synaptopodin staining in normal kidney tissue as positive control, and (f) synaptopodin staining in normal bladder tissue as negative control.

In total, 56 patients were recruited. The diagnoses at time of urine collection were as follows: preeclampsia (*n* = 28), eclampsia (*n* = 1), chronic hypertension (*n* = 3), gestational hypertension (*n* = 6), Type I DM (*n* = 1), Type II DM (*n* = 1), gestational DM (*n* = 4), connective tissue disorder (*n* = 1), marginal previa (*n* = 1), chromosomal anomaly (*n* = 1), and uncomplicated pregnancy (*n* = 9). The clinical characteristics of all subjects are described in Table 1. Podocyturia (Figures 1(a) and 1(b)) was present in 11 out of 29 (38%) patients with preeclampsia/eclampsia, 3 out of 9 (33%) with chronic and gestational HTN, and 3 out of 6 (50%) with gestational DM and Type I/II DM (Table 2). Among patients categorized as “other”, 2 (marginal previa and chromosomal anomaly) out of 3 patients exhibited podocyturia (66%). In contrast, 0 out of 9 patients (0%) with uncomplicated pregnancies demonstrated podocyturia. Based on these findings, we calculated the sensitivity and specificity of podocyturia for preeclampsia to be 38% and 70%, as compared to women of HTN of any type, in whom it was 33% and 66%, respectively (Table 3). The sensitivity and specificity for DM of any type were 50% and 68%, respectively. The positive predictive value was 57% for preeclampsia, as compared with 15% for both HTN of any type and DM of any type. The negative predictive value, however, was poor for preeclampsia at 51%, as compared with 83% and 91% for HTN of any type and DM of any type, respectively.

## 4. Discussion

Podocyturia is well described in many glomerular diseases such as Type I DM, IgA nephropathy, lupus nephritis, and membranous nephropathy [5, 6]. Though endothelial injury is thought to be the main lesion in preeclampsia, more recently, derangements of the podocyte with downregulation of selected podocyte-specific proteins in renal biopsies and presence of nephrin and podocalyxin (podocyte specific proteins) in the urine have been described [2, 7]. In this study, we sought to describe the presence of podocyturia in preeclampsia and other high risk pregnancy states using the podocyte marker synaptopodin for identification. We found that podocyturia was neither sensitive nor specific in making this diagnosis. These results are in contrast to an earlier study by Garovic et al. [2]. Though the exact reason for this discrepancy is unclear, immunofluorescent staining of podocyte-specific proteins, in general, does not appear to be an accurate tool to identify podocytes, as these podocytes may be parietal in origin [8] and may also be apoptotic [9]. Thus, the utility of urinary podocytes to detect ongoing glomerular damage in women with preeclampsia as previously suggested [10] is unclear. Furthermore, our study showed presence of podocyturia in other high-risk pregnancy states such as DM (gestational or Type I/II), HTN (gestational or chronic). This finding is not unexpected since podocyturia and urinary podocyte mRNA have been described in nonpregnant diabetic patients [6] and in nonpregnant hypertensive patients [11] respectively. Interestingly, the number of urinary podocytes has also shown a statistically significant correlation with blood pressure but not proteinuria in preeclampsia [12].

Though podocyturia might help shed light on the pathophysiology of preeclampsia, we feel that its clinical utility is limited. There are several limitations in relying on the podocytes as biomarkers. Identifying podocytes in the urine is highly laborious and is technically challenging. Both cytospin methods (as was performed in this study) and cultivation of podocytes in culture are fraught with difficulties and are not cost-effective. It may not be easy to eliminate podocyte cell debris when counting podocytes from cytospin specimen of fresh sediment [9]. Growing urinary podocytes in cell culture, on the other hand, are frequently limited by bacterial or fungal contamination as the urine may not have been collected under sterile conditions. Furthermore, these cells may proliferate, undergo apoptosis, or not attach to the culture dish, thereby falsely representing the true podocyte count [9]. Interobserver bias poses yet another obstacle as it requires a highly trained cytologist to correctly identify these cells.

Since clinical guidelines are available to diagnose preeclampsia, some have questioned whether the addition of a urinary marker is necessary. We feel that the utility of this marker becomes important when the diagnosis of preeclampsia is in question and when the clinical scenario is complicated by the presence of preexisting HTN, DM, or other glomerular diseases such as lupus nephritis. In those cases, the treatment would gear towards the underlying condition, in addition to supportive care. Thus, a specific marker that is discovered through our understanding of the pathophysiology of preeclampsia remains crucial. Whether podocyturia is that marker remains unanswered.

## 5. Limitations of the Study

The major limitation of our study is the small sample size. Our goal was to confirm the previously shown high sensitivity and specificity of podocyturia in preeclampsia. Though a larger sample size would be ideal, we feel that of our total sample size of 56 with 29 patients diagnosed with preeclampsia/eclampsia, and 18 patients with other high-risk diagnoses, is an adequate sample size to demonstrate that podocyturia lacks sensitivity and specificity to be a diagnostic marker of preeclampsia. 

## 6. Conclusions

We discovered that podocyte loss is present not only in preeclampsia but in other high risk pregnancy states. In addition, podocyturia was not found in a majority of patients diagnosed with preeclampsia. We realize our finding of a relatively low sensitivity and specificity is not conclusive. However, our findings raise an important note of caution of relying on the limited findings in the literature regarding the predictive value of podocyturia in preeclampsia and encourage larger studies. Given our current knowledge of the complex pathophysiology of the disease such as the significant role of vascular endothelial growth factor (VEGF) signaling in maintaining a healthy endothelium and cross talk between the podocyte and vascular endothelial cell [13], it is unlikely that any single test or cell type will be able to predict preeclampsia. A panel of biomarkers reflective of this complexity may be ideal for diagnosis.

## Figures and Tables

**Table 1:** Patient Characteristics.

Variable	Normal (9)	Preeclampsia/eclampsia (29)	^ a^HTN-chronic/gestation (9)	^ b^DM-Type I/II/Gestational (6)	^ c^Other (3)	*P* value
Maternal age (yr)	29.8 ± 4.4	27.9 ± 4.5	29.7 ± 4.3	28.5 ± 3.5	30.7 ± 6.9	0.52
Gestational age (wk)	37.4 ± 1.5	32.1 ± 2.7	32.3 ± 2.5	27.8 ± 6.9	26.6 ± 1.5	<0.0001
Systolic blood pressure (mmHg)	114.5 ± 12.0	156.6 ± 17.8	158.5 ± 27.0	120.8 ± 6.3	126.5 ± 6.6	<0.0001
Diastolic blood pressure (mmHg)	72.4 ± 7.2	95.0 ± 8.25	92.6 ± 8.0	75.1 ± 3.7	68 ± 9.8	<0.0001
^ d^Proteinuria (mg/24 hr)	149 ± 0	1099 ± 973	150 ± 3.5	118 ± 50	109 ± 68	<0.0001

^a ^HTN: hypertension.

^b ^DM: diabetes mellitus.

^c ^Other: diagnoses marginal previa (1), chromosomal anomaly (1), connective tissue disorder (1).

^d ^24-hour proteinuria extrapolated from a urine dipstick value as described in methods section.

**Table 2:** Presence of podocyturia in various pregnancy categories.

	Podocyte positive	Podocyte negative	% Positive
Preeclampsia/eclampsia (29)	11	18	38%
HTN-Gestational/chronic (9)	3	6	33%
DM: any type (6)	3	3	50%
^ a^Others (3)	2	1	66%
^ b^Controls (9)	0	9	0%

^a^Other: diagnoses of marginal previa (1), chromosomal anomaly (1), connective tissue disorder (1).

^b^Controls: uncomplicated pregnancies.

**Table 3:** Test characteristics for podocyturia.

	Sensitivity	Specificity	Positive predictive value	Negative predictive value
Preeclampsia/eclampsia	38%	70%	57%	51%
HTN (any type)	33%	66%	15%	83%
DM (any type)	50%	68%	15%	91%
